# Efficient Removal of Hazardous P-Nitroaniline from Wastewater by Using Surface-Activated and Modified Multiwalled Carbon Nanotubes with Mesostructure

**DOI:** 10.3390/toxics12010088

**Published:** 2024-01-19

**Authors:** Tzong-Horng Liou, Jyun-Jie Huang

**Affiliations:** 1 Department of Chemical Engineering, Ming Chi University of Technology, 84 Gungjuan Rd., Taishan District, New Taipei City 24301, Taiwan; 2 Department of Chemical and Materials Engineering, Chang Gung University, 259 Wenhua 1st Rd., Guishan District, Taoyuan City 33302, Taiwan

**Keywords:** carbon nanotube, activation, carbon–oxygen functional group, p-nitroaniline, recycling

## Abstract

P-nitroaniline (PNA) is an aniline compound with high toxicity and can cause serious harm to aquatic animals and plants. Multiwalled carbon nanotubes (MWCNTs) are a multifunctional carbon-based material that can be applied in energy storage and biochemistry applications and semiconductors as well as for various environmental purposes. In the present study, MWCNTs (CO_2_–MWCNTs and KOH–MWCNTs) were obtained through CO_2_ and KOH activation. ACID–MWCNTs were obtained through surface treatment with an H_2_SO_4_–HNO_3_ mixture. Herein, we report, for the first time, the various MWCNTs that were employed as nanoadsorbents to remove PNA from aqueous solution. The MWCNTs had nanowire-like features and different tube lengths. The nanotubular structures were not destroyed after being activated. The KOH–MWCNTs, CO_2_–MWCNTs, and ACID–MWCNTs had surface areas of 487, 484, and 80 m^2^/g, respectively, and pore volumes of 1.432, 1.321, and 0.871 cm^3^/g, respectively. The activated MWCNTs contained C–O functional groups, which facilitate PNA adsorption. To determine the maximum adsorption capacity of the MWCNTs, the influences of several adsorption factors—contact time, solution pH, stirring speed, and amount of adsorbent—on PNA adsorption were investigated. The KOH–MWCNTs had the highest adsorption capacity, followed by the CO_2_–MWCNTs, pristine MWCNTs, and ACID–MWCNTs. The KOH–MWCNTs exhibited rapid PNA adsorption (>85% within the first 5 min) and high adsorption capacity (171.3 mg/g). Adsorption isotherms and kinetics models were employed to investigate the adsorption mechanism. The results of reutilization experiments revealed that the MWCNTs retained high adsorption capacity after five cycles. The surface-activated and modified MWCNTs synthesized in this study can effectively remove hazardous pollutants from wastewater and may have additional uses.

## 1. Introduction

The discharge of wastewater from dye industries has caused water pollution and recently gained global attention. P-nitroaniline (PNA) is an important intermediate, which is used in the pesticide, antioxidant, fuel additive, and dye industries [1]. In even small quantities, effluents containing toxic substances can have detrimental effects on aquatic environments [2]. Several physicochemical techniques—including adsorption, photodegradation, bio-decomposition and electrochemical treatment—have been developed for treating PNA-containing wastewater [3]. Of these techniques, adsorption is considered the most efficient approach due to its low cost and simplicity of operation, even for treating large quantities of wastewater. Various analytical techniques—including high-performance liquid chromatography, gas chromatography, gas chromatography–mass spectrometry, and liquid chromatography–mass spectrometry—have been employed to measure dye concentrations. These methods have several drawbacks, such as complexity of operation leading to slow results and high costs, which can limit the efficiency of analysis. An alternative analytical technique for measuring dye concentrations is ultraviolet–visible (UV-Vis) spectroscopy, which is cheap and user-friendly.

A carbon nanotube (CNT) is a cylindrical object with a radius smaller than 100 nm and a length longer than 20 μm [4]. CNTs can be categorized as single-walled CNTs or multiwalled CNTs (MWCNTs). Single-walled CNTs consist of only one graphene sheet, whereas MWCNTs consist of more than one graphene sheet [5]. Hexagonal arrays of carbon atoms grow on the surface of CNTs, meaning that CNTs are highly conductive and mechanically strong. CNTs can be used in CO_2_ adsorption [6], H_2_ evolution [7], composite catalysis [8], and electrode materials [9]. They interact with organic molecules; these interactions include hydrophobic interactions, van der Waals forces, electrostatic forces, π–π stacking, and hydrogen bonding [10]. CNTs have several advantages for capturing organic and inorganic substances, such as high adsorption capacity and high equilibrium rates. The entwinement of CNTs in water-based media reduces the surface area of the CNTs, thereby limiting their adsorption capacity. To address this issue, CNTs can be surface-activated and modified in an inert gas environment to create microporous and mesoporous structures. These modifications effectively increase the nanotubes adsorption capacity. CNTs can be activated either physically or chemically. Physical activation involves using a carbon precursor in the presence of CO_2_ or a gas stream and then performing carbonization at high temperature [11]. Chemical activation involves using KOH, NaOH, H_3_PO_4_, or ZnCl_2_ as an activating agent for an activation reaction [12]. Surface-activated and modified CNTs are particularly suitable for use in hydrogen adsorption [13], methane storage devices [14], supercapacitors [15], catalyst supports [16] as well as wastewater treatment [17].

Several adsorbents—such as covalent organic frameworks (COFs) and metal-organic frameworks (MOFs) [18,19,20,21], graphene nanosheets, CNTs, and activated carbon (AC)—have been widely used in the treatment of dye wastewater. More et al. [22] used a chemical reduction method to fabricate Co_3_O_4_–MWCNT composites with a nanowire-like appearance and various pore diameters. These composites exhibited high efficiency in catalytic adsorption of coracyl yellow dye and reduction of PNA concentration. Zhao et al. [23] developed core–shell magnetic MWCNTs through tailored graft polymerization by using polyethyleneimine. The high positive charge density of polyethyleneimine effectively increased the nanotubes’ ability to adsorb anionic dyes, resulting in maximum adsorption capacities for methyl orange, conge red, and methyl blue of 935, 1006, and 1449 mg/g, respectively. Samiyammal et al. [24] synthesized AC from cashew nut shells through KOH activation; the highest surface area, 407.80 m^2^/g, was achieved for a KOH ratio of 50%, temperature of 600 °C, and duration of 2 h. The maximum capacity for adsorption of brilliant green was 243.90 mg/g. Yu et al. [25] synthesized AC by using cattail biomass and CO_2_ activation, and their AC had a pore volume and surface area of 0.33 cm^3^/g and 441.2 m^2^/g, respectively, and a maximum capacity for adsorption of malachite green of 210.8 mg/g. Their AC also exhibited excellent electrochemical performance characteristics, with a specific capacitance value of 126.5 F/g. AC has a large surface area that provides numerous adsorption sites. However, the traditional activation process for AC results in microporosity, leading to high mass transfer resistance [26,27]. MWCNTs have a hollow structure that provides high mesoporosity and ease of modification. MWCNTs activated with CO_2_ and KOH have low density, a large pore volume, and a large surface area, making them exceptional nanoadsorbents for capturing organic and inorganic pollutants. The mesoporous structure within activated MWCNTs provides several advantages, including rapid mass transfer and high degradation activity.

This study synthesized three types of CNTs, namely ACID–MWCNTs, CO_2_–MWCNTs, and KOH–MWCNTs, by activating and modifying the surface of MWCNTs. First, ACID–MWCNTs were synthesized through surface treatment of CNTs by using an H_2_SO_4_–HNO_3_ mixture. Second, CO_2_–MWCNTs and KOH–MWCNTs were synthesized through physical and chemical activation of CNTs, with CO_2_ and KOH employed as the activating agent, respectively. The purpose of the surface activation and modification was to improve the adsorption capacity of the nanotubes by creating more adsorption sites for the capture of organic molecule. This study is the first to compare the efficacy of these three types of MWCNTs in the removal of PNA from wastewater. The physicochemical characteristics of the synthesized MWCNTs—such as their crystalline phase, functional groups, elemental composition, thermal stability, graphitization, pore characteristics, and surface and internal morphologies—were extensively examined. Factors that influence the efficiency of PNA removal—contact time, stirring speed, pH, and adsorbent amount—were also examined to determine their effects on the adsorption capacity. The interaction between PNA and MWCNTs and the adsorption mechanism were investigated through isotherm and kinetics experiments. Reutilization tests were also conducted to evaluate the reusability of the synthesized MWCNTs.

## 2. Materials and Methods

### 2.1. Materials

Fe_2_O_3_ was purchased from Acros Organic (Morris Plains, NJ, USA). PNA (C_6_H_6_N_2_O_2_, molecular weight = 138.12 g/mol), H_2_SO_4_, HNO_3_, KOH, C_2_H_5_OH, NaOH, and HCl were purchased from Merck (Gernsheim, Germany). CH_4_, CO_2_, N_2_, and an air mixture (21% O_2_ and 79% N_2_) were purchased from Sun Fu Co. (Taipei, Taiwan).

### 2.2. MWCNT Synthesis

MWCNTs were synthesized using catalytic chemical vapor deposition [28,29]. CH_4_ was employed as a carbon source, and Fe_2_O_3_ was employed as a catalyst. The reaction temperature was set to 800–1000 °C. The CNTs were grown in a horizontal reactor that consisted of a quartz tube with a diameter of 5 cm and a length of 100 cm. After the reaction, the synthesized CNTs were immersed in HCl solution to remove catalyst residues. The pristine nanotubes are referred to as COM–MWCNTs.

### 2.3. Modification of MWCNTs

ACID–MWCNTs, CO_2_–MWCNTs, and KOH–MWCNTs were synthesized as follows:

(1) ACID–MWCNTs [30]: 1.0 g of MWCNTs was dispersed in 40 mL of H_2_SO_4_–HNO_3_ solution (molar ratio = 3:1). The acidic solution was dropped slowly with 10 mL of anhydrous alcohol to increase the hydrophilicity of the carbon solid. The solution was subsequently heated to 70 °C and stirred continuously for 24 h, after which it was washed in water until it became neutral to remove any acidic residues. The solution then underwent drying and grinding processes until an acid-modified MWCNT solid was obtained. The obtained solid is referred to as the ACID–MWCNTs.
(2) CO_2_–MWCNTs [31]: 1.0 g of MWCNTs was dried at 100 °C for 24 h in an air oven. The dried solid was placed on a ceramic boat and then inserted into a quartz tubular reactor. The carbonaceous solid was subsequently heated at 850 °C for 4 h. CO_2_ was employed as the reaction gas. The flow rate was 60 mL/min. The obtained solid is referred to as the CO_2_–MWCNTs.
(3) KOH–MWCNTs [32,33]: 4.0 g of KOH was dissolved in 50 mL of distilled water. MWCNTs (1.0 g) were then added to the solution. The solution was subsequently stirred continuously at 120 °C, left to dry for 24 h, and then heated to 800 °C (at a rate of 10 °C/min) for 1 h under pure N_2_ atmosphere. The obtained solid was rinsed with HCl solution (1.0 M). Afterward, the solid was washed with deionized water and dried. The solid is referred to as the KOH–MWCNTs.

### 2.4. Adsorption Experiment

PNA adsorption on the nanoadsorbents (COM–MWCNTs, ACID–MWCNTs, CO_2_–MWCNTs, and KOH–MWCNTs) was investigated using a bath apparatus. First, 10 mg of MWCNTs was added to 100 mL of PNA solution (PNA concentration = 150 mg/L). The solution was stirred for 2 h to ensure adsorption equilibrium. The effects of other factors—contact time, solution pH, stirring speed, and CNT amount—on the nanotubes’ adsorption capacity were investigated. The PNA concentration was measured using a UV-Vis spectrophotometer (Genesys, Thermo Electron Corporation, Waltham, MA, USA). Adsorption capacity *q_t_* was calculated as follows:

(1) $$q_{t}(mg/g)=\frac{\left(C_{0}-C\right)V}{W}$$

where *V* is the volume of the PNA solution (L), *W* is the CNT amount (g), and *C*_0_ and *C* are the PNA concentrations at baseline and at time *t*, respectively.

The reusability of the synthesized MWCNTs was assessed. First, the adsorption of PNA on the MWCNTs was conducted under optimal conditions. Once the adsorption equilibrium had been reached, desorption experiments were conducted by subjecting the MWCNTs to thermal treatment at 400 °C in N_2_ atmosphere to achieve the maximum desorption efficiency. Adsorption–desorption experiments were performed at least five times, and PNA concentrations were measured using a UV-Vis spectrophotometer.

### 2.5. Characterization of CNT Materials

The pore volume, surface area, and pore diameter of the MWCNTs were evaluated using an ASAP 2020 adsorption analyzer (Micromeritics, Norcross, GA, USA) at −196 °C. An X-ray diffractometer (X’pert Pro System, PANalytical, Malvern, UK) with Cu Kα radiation was employed to observe the crystalline phase of the MWCNTs. A field-emission scanning electron microscope (JEOL JSM-6700F, Akishima, Tokyo, Japan) and a transmission electron microscope (JEOL JEM-1200CX II, Akishima, Tokyo, Japan) were used to examine their morphological features. A Fourier transmission infrared spectroscope (FTIR-8300, Shimadzu, Nakagyo-ku, Kyoto, Japan) was employed to examine the functional groups of the MWCNTs. Graphitization was assessed using a confocal Raman spectroscope (Renishaw, Gloucestershire, UK) with 632 nm He–Ne laser excitation. The stability of the MWCNTs before and after modification was examined using a thermogravimetric analyzer (Mettler Toledo, OH, USA, model TGA/SDTA851e). The surface elements on the MWCNTs were analyzed using an X-ray photoelectron spectroscope (Esca Lab 250Xi, Thermo Scientific, Waltham, MA, USA). The C1s peak at 284.60 eV was employed to calibrate the binding energy. The amounts of metallic impurities in the MWCNTs were determined using an inductively coupled plasma–mass spectrometer (ICP-MS) (Konton Plasmakon, Eching, Germany, model S-35).

## 3. Results and Discussion

### 3.1. Characterization of Nanotubes

**Figure 1:**
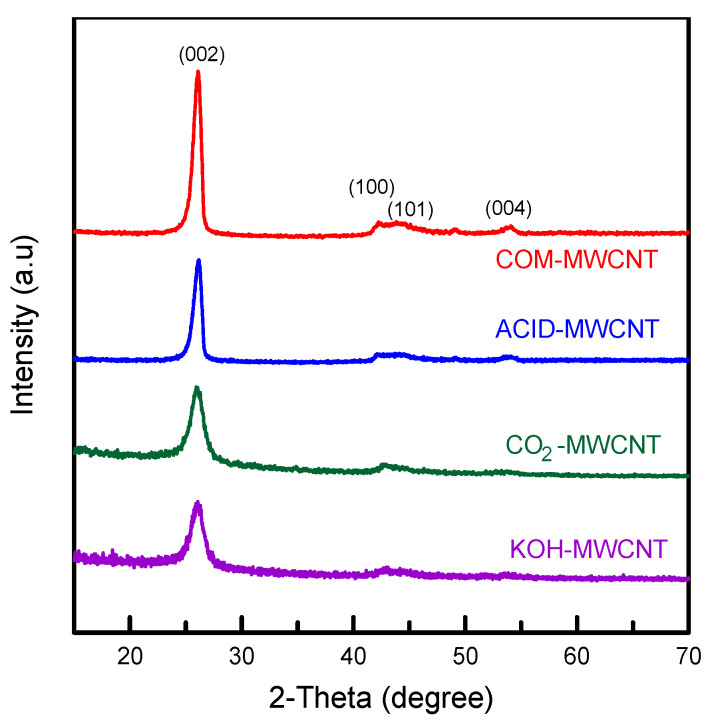
XRD patterns of MWCNTs before and after modification.

Figure 1 depicts the X-ray diffraction patterns of the MWCNTs. The COM–MWCNT sample was not treated with chemical reagents. The peak at 2θ = 25.9° corresponds to diffraction from the (002) plane, characteristic of hexagonal graphite structures [34]. The signals detected at 2θ = 43°, 45°, and 53.5° correspond to the (100), (101), and (004) planes, characteristic of graphite structures [35]. For the ACID–MWCNT, CO_2_–MWCNT, and KOH–MWCNT samples, peaks corresponding to the (002), (100), (101), and (004) planes were again observed. However, the peaks in the spectra of the ACID–MWCNT, CO_2_–MWCNT, and KOH–MWCNT samples were less intense than those in the spectrum of the COM–MWCNT sample. Similar characteristics were also observed by Zhang and Chen [36]. The hexagonal frame of the CNTs’ graphite structure was evidently not destroyed by surface activation and modification. Table 1 shows the purity of the MWCNTs before and after modification [37]. These nanotubes have the low concentration of metallic impurities. It can be ascribed to metals dissolving in the acidic solution after the HCl treatment. These metals were easily removed by leaching and filtration processes [12]. Moreover, CO_2_- or KOH-activated samples had lower metal elements than pristine nanotubes. This decrease in impurities may be because metals were carried by the vaporization of carbonaceous matter during activation.

**Figure 2:**
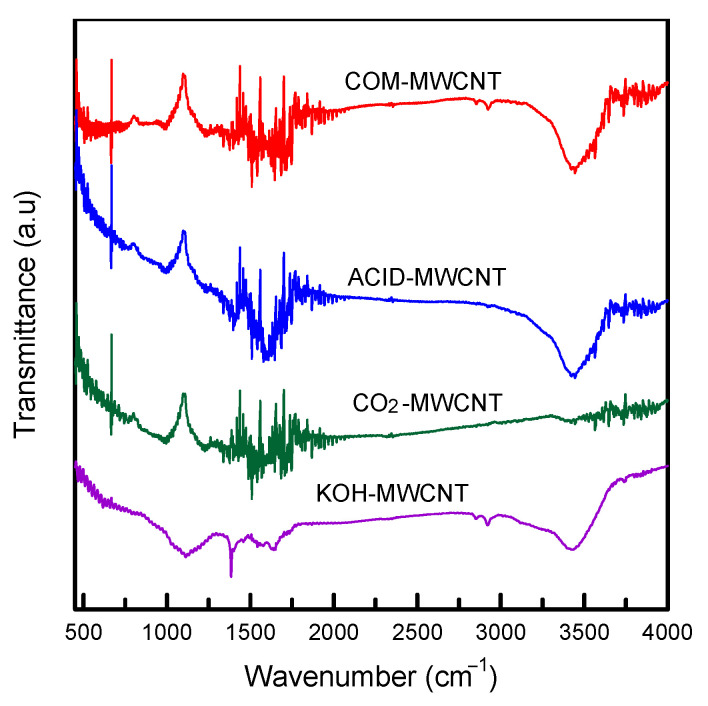
FTIR spectra of MWCNTs before and after modification.

Functional groups were examined using Fourier transmission infrared spectroscopy (Figure 2). For all samples, the peak at 3400–3500 cm^−1^ corresponds to stretching of hydroxyl groups (OH) [38]. The band at 2920 cm^−1^ corresponds to symmetric and asymmetric stretching of C–H groups. The peak at 1040 cm^−1^ corresponds to stretching of –COOH. In the case of the CO_2_–MWCNTs and KOH–MWCNTs, the peaks at 1375, 1625, and 1720 cm^−1^ represent C–O, C=C, and C=O stretching, respectively [10]. These O-containing functional groups can effectively combine organic molecules through electrostatic attraction, van der Waals forces, and π–π interactions [39]. These groups are thus favorable for the removal of contaminants.

**Figure 3:**
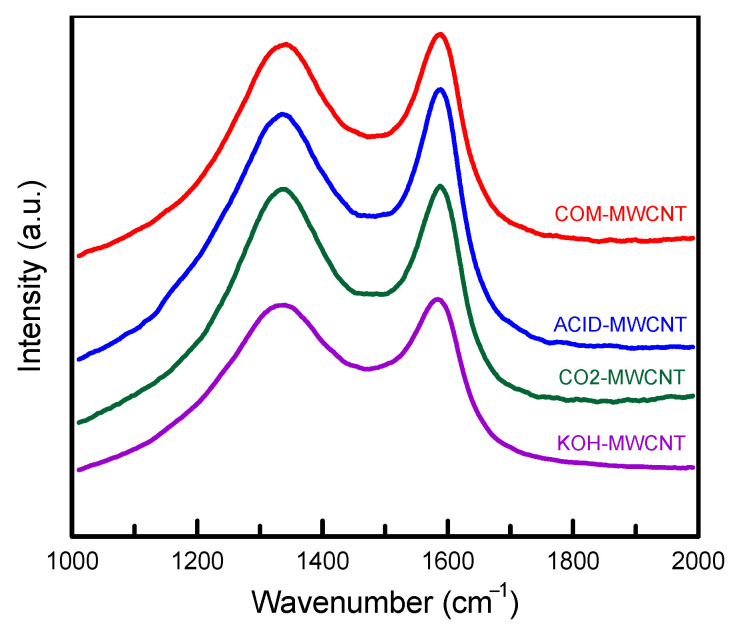
Raman spectra of MWCNTs before and after modification.

Figure 3 displays the Raman spectra of the unmodified and modified MWCNTs. The first peak at 1300–1340 cm^−1^ was attributable to disorder and defects (D band), and the second peak at 1570–1620 cm^−1^ was attributable to G band features [40]. The ratio of D band intensity to G band intensity (I_D_/I_G_) indicates the ratio of sp^3^ to sp^2^ carbon atom [41]. The intensity ratio was highest for the CO_2_–MWCNTs (0.9859), followed by the KOH–MWCNTs (0.9606), COM–MWCNTs (0.9445), and ACID–MWCNTs (0.9348). More sp^3^ carbon atoms were observed on the activated samples (CO_2_–MWCNTs and KOH–MWCNTs) than on the nonactivated samples. The activated MWCNTs displayed a high degree of oxidation, which suggested higher adsorption capacity [42].

**Figure 4:**
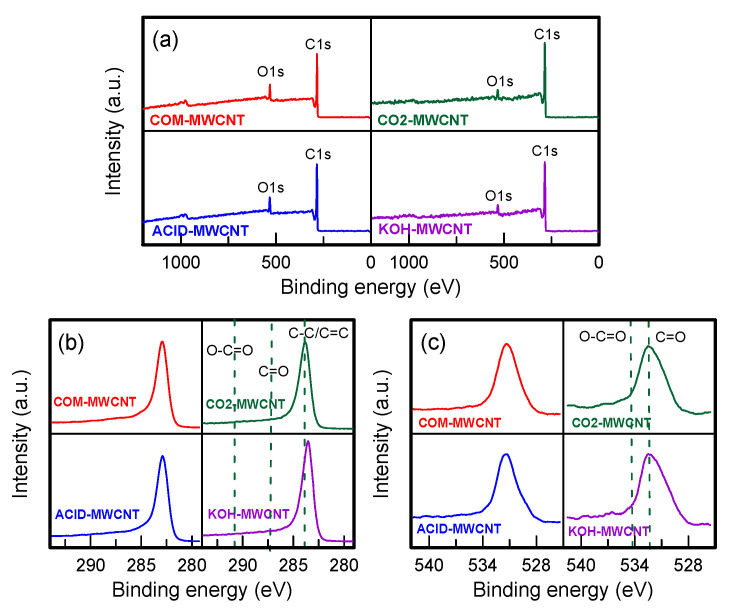
XPS analysis of MWCNTs: (**a**) wide survey spectra, (**b**) C1s, and (**c**) O1s.

The elements distributed on the surface of the unmodified and modified MWCNTs were inspected using X-ray photoelectron spectroscopy (Figure 4). Wide survey spectra confirmed that C and O were the main elements (Figure 4a) [43]. In the C1s spectra (Figure 4b), the peak at approximately 284.0 eV was attributable to C–C and C=C groups, which came from the diamond-like sp^3^ carbon and graphite-like sp^2^ carbon, respectively. According to Zhao et al. [41], peaks at 287.5 and 291.0 eV are attributable to C=O and O–C=O bonds, respectively. However, the two peaks in our spectra were very weak. The O1s spectra (Figure 4c) contained peaks at 532.3 and 533.7 eV. These were associated with C=O and O–C=O bonds, respectively [44]. The C–O functional groups in the MWCNTs would aid the π–π interaction between PNA molecules and the MWCNTs, thereby increasing the MWCNTs’ adsorption activity.

**Figure 5:**
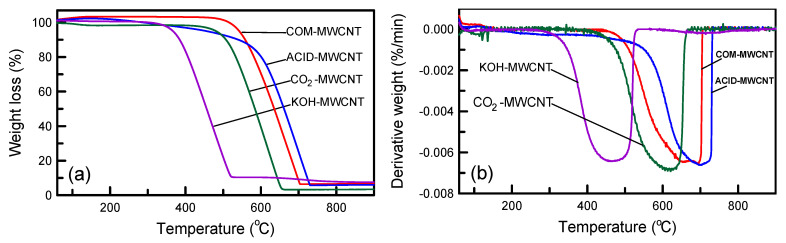
(**a**) TG and (**b**) DTG curves of MWCNTs.

The thermal stability of the MWCNTs before and after modification was examined using thermogravimetric analysis (Figure 5). For all MWCNTs, weight loss could be divided into two zones (Figure 5a). The initial weight loss at 25–150 °C was due to moisture loss. The greatest weight loss at 150–800 °C may have been attributable to decomposition of the carbonaceous matters. The presence of ash residue (3.4–7.5 wt%) indicated that the MWCNTs did not completely decompose during oxidation. The CO_2_–MWCNTs had the highest purity. Peaks indicating the maximum rate of thermal decomposition are visible in Figure 5b. The peak temperatures for the KOH–MWCNTs, CO_2_–MWCNTs, COM–MWCNTs, and ACID–MWCNTs were 467, 621, 692, and 705 °C, respectively. The ACID–MWCNTs had the highest thermal stability. The KOH–MWCNTs and CO_2_–MWCNTs had lower thermal decomposition temperatures than did the COM–MWCNTs and ACID–MWCNTs, possibly because the KOH–MWCNTs and CO_2_–MWCNTs were more porous.

### 3.2. Surface Area and Pore Features

**Figure 6:**
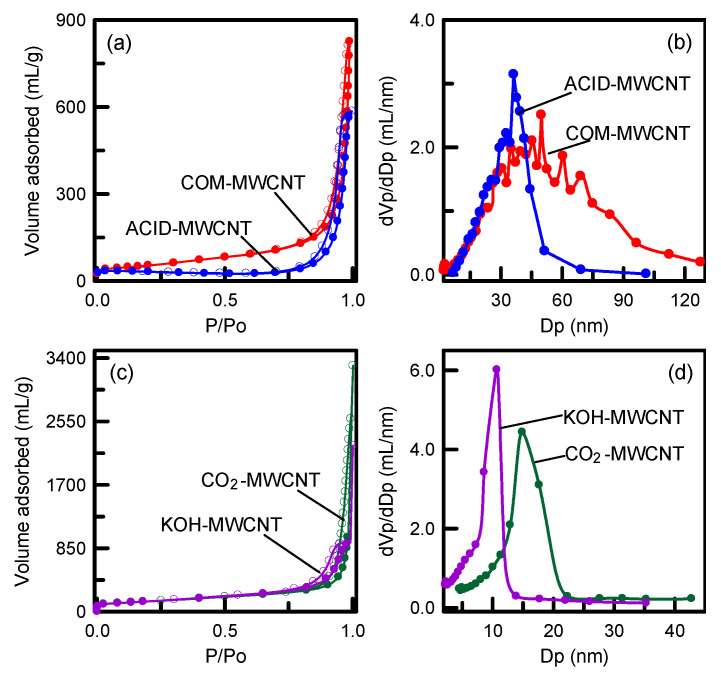
(**a**,**c**) N_2_ sorption isotherm and (**b**,**d**) pore size distribution of MWCNTs.

The pore structure and surface area of the CNTs before and after modification were examined using an N_2_ sorption experiment. As shown in Figure 6a,c, similar loops were obtained for all types of MWCNTs, indicating a type-IV isotherm (International Union of Pure and Applied Chemistry classification) [45]. The four carbons containing an H3 hysteresis loop were typical mesoporous materials. The mesostructure came from tubular channels and the entanglement of nanotubes. The N-adsorbed volumes of the CO_2_–MWCNTs and KOH–MWCNTs were much higher than those of the COM–MWCNTs and ACID–MWCNT. The high adsorption volumes were mainly a result of the vaporization of MWCNT tissues during activation [46]. The nanotubes’ pore size distributions are presented in Figure 6b,d. The pore size distribution of the COM–MWCNTs was broad, covering sizes between 2 and 120 nm. Intertube spaces formed through aggregation of long tubes may have led to this broad pore size distribution [36]. By contrast, the pore size distributions of the ACID–MWCNTs, CO_2_–MWCNTs, and KOH–MWCNTs were narrow, indicating a uniform pore structure. The average pore diameters of the ACID–MWCNTs, CO_2_–MWCNTs, and KOH–MWCNTs were 36.29, 14.87, and 10.70 nm, respectively. Notably, the KOH–MWCNTs had smaller pores than did the CO_2_–MWCNTs. Chemical activation may have more drastic effects than does physical activation. The surface area, pore volume, and pore fraction values are listed in Table 2. The pristine nanotubes (COM–MWCNTs) had a surface area of 189 m^2^/g and a pore volume of 1.039 cm^3^/g. The surface area and pore volume of the ACID–MWCNTs, which were treated with H_2_SO_4_–HNO_3_, were 80 m^2^/g and 0.871 cm^3^/g, respectively. The surface areas and pore volumes of the CO_2_–MWCNTs and KOH–MWCNTs, which were physically and chemically activated, were 484 m^2^/g and 1.321 cm^3^/g, respectively, and 487 m^2^/g and 1.432 cm^3^/g, respectively. An increase in mesoporous volume may cause an increase in the textural parameters of activated samples. The KOH–MWCNTs had the highest surface area and pore volume, consisting with the observation in Figure 6a,c. The mesopore fraction of the ACID–MWCNTs (91.50%) was lower than that of the COM–MWCNTs (98.84%), indicating that strong acid etching on the carbon surface resulted in the formation of a microporous structure. The mesopore fractions of the CO_2_–MWCNTs and KOH–MWCNTs were 99.32% and 97.63%, respectively. The physically activated MWCNTs had the highest mesoporosity.

### 3.3. Surface Morphology

**Figure 7:**
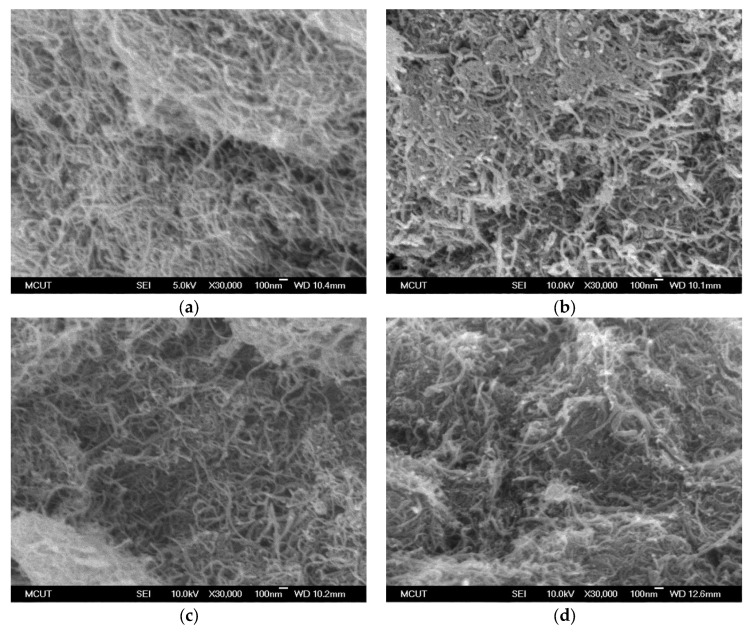
FE-SEM images of nanotubes: (**a**) COM-MWCNT, (**b**) ACID-MWCNT, (**c**) CO_2_-MWCNT, and (**d**) KOH-MWCNT.

The morphologies of the MWCNTs before and after modification were examined using scanning electron microscopy (Figure 7). Compared with the diameter of the pristine MWCNTs (Figure 7a), the MWCNTs modified using H_2_SO_4_–HNO_3_ was coarser (Figure 7b), indicating that the MWCNTs were inflated by the acidic treatment. After activation (Figure 7c,d), the MWCNTs were shorter, indicating that the MWCNTs were sectioned into smaller MWCNTs as the carbon skeleton was etched with CO_2_ or KOH [36]. The drastic gasification during physical and chemical activation resulted in pore opening on the inner surface of MWCNTs, increasing the microporous and mesoporous volumes and thereby increasing the nanotubes’ adsorption capacity.

**Figure 8:**
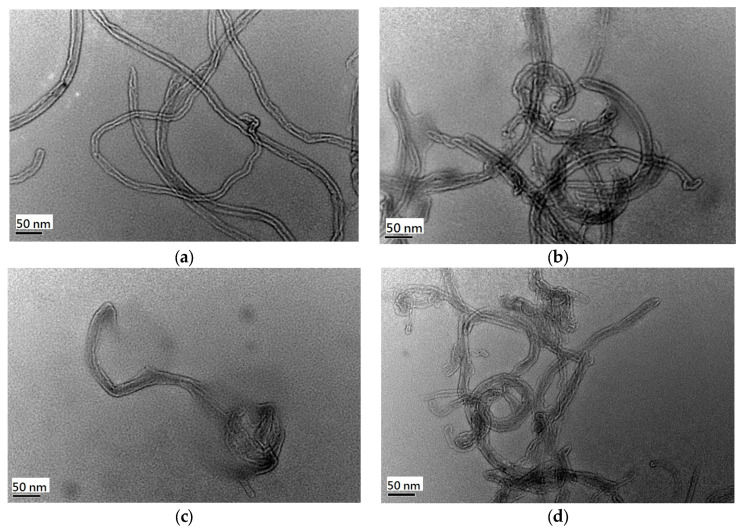
TEM images of nanotubes: (**a**) COM-MWCNT, (**b**) ACID-MWCNT, (**c**) CO_2_-MWCNT, and (**d**) KOH-MWCNT.

The internal morphologies of the MWCNTs before and after modification were examined using transmission electron microscopy (Figure 8). All of the MWCNTs were hollow and tubular. The acid and alkali treatments did not affect the internal morphologies of the MWCNTs [47]. The COM–MWCNT had a glossy and smooth surface (Figure 8a). The acidified nanotubes had a relatively coarse surface (Figure 8b). The ACID–MWCNTs were tied in knots and twisted, resulting in less surface area being available than for the pristine nanotubes. The observation consisted with the results of surface area analysis in Table 2. The activated nanotubes were shorter than the nonactivated nanotubes (Figure 8c,d) The surface of the activated nanotubes was coarser than that of the pristine nanotubes due to the drastic oxidation reaction during activation. These conditions are helpful for increasing the surface area of the nanotubes (Table 2).

### 3.4. Adsorption Performance of CNTs

**Figure 9:**
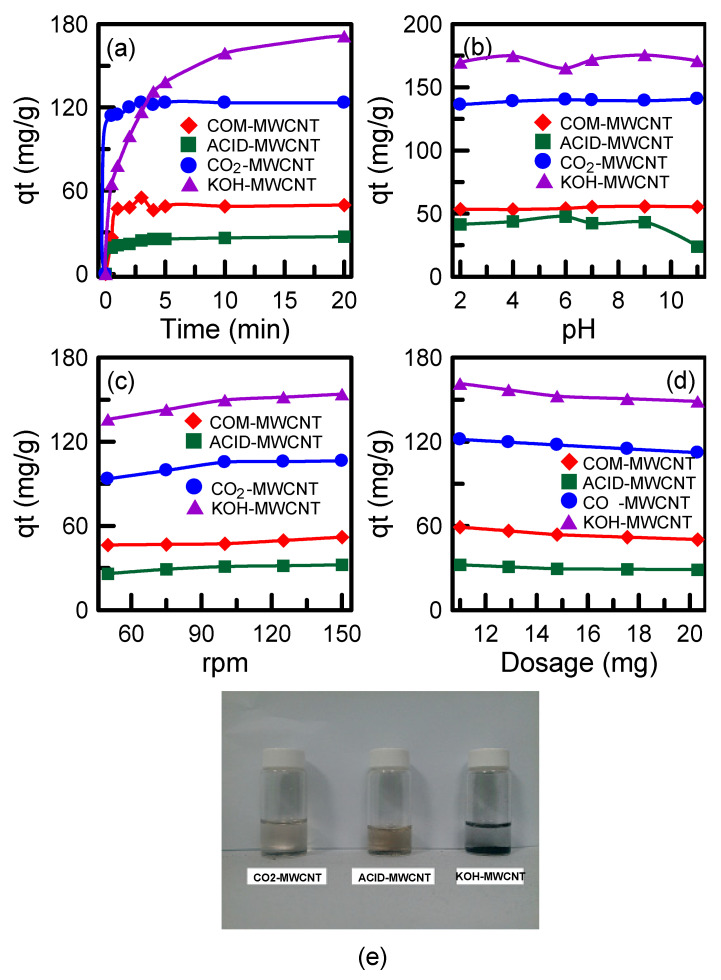
Effect of different parameters on the PNA adsorption onto MWCNTs: (**a**) nanotube types, (**b**) solution pH, (**c**) stirring speed, (**d**) amount of nanotube, and (**e**) optical photographs of residual solutions.

The PNA adsorption capabilities of the COM–MWCNTs, ACID–MWCNTs, CO_2_–MWCNTs, and KOH–MWCNTs were evaluated. The ACID–MWCNTs had lower adsorption capacity than did the COM–MWCNTs, indicating that acid treatment did not improve the nanotubes’ adsorption capacity (Figure 9a). Corrosion due to acid on the nanotube surface may cause the collapse of pore walls. The reduction in surface area and pore volume for the ACID–MWCNT sample (Table 2) resulted in lower adsorption capability. The CO_2_–MWCNTs and KOH–MWCNTs had high adsorption capabilities compared with the COM–MWCNTs because their surface area and pore volume were higher than those of the pristine nanotubes. In addition, the presence of oxygen-containing functional groups in the activated nanotubes (Figure 2) promoted the interactions between PNA and the nanotubes, leading to an enhancement of PNA adsorption [48]. The KOH–MWCNTs had higher adsorption capacity than did the CO_2_–MWCNTs. This was because the KOH–MWCNTs had a higher pore volume (Table 2). Despite the higher adsorption capacity of the KOH–MWCNTs, the CO_2_–MWCNTs exhibited adsorption ability significantly higher than that of the KOH–MWCNTs in the initial 2 min, possibly because the pores of the KOH–MWCNTs were narrower than those of the CO_2_–MWCNTs (Table 2), which caused long-distance diffusion of PNA into the nanotube pores. The relatively large diameter of pores in the CO_2_–MWCNTs meant that the pores did not become blocked, which was beneficial to PNA adsorption. The maximum adsorption capacities of the ACID–MWCNTs, COM–MWCNTs, CO_2_–MWCNTs, and KOH–MWCNTs were 26.8, 49.6, 123.4, and 171.3 mg/g, respectively. The results indicate that activated nanotubes are excellent adsorbent materials for recovering organic contaminants, such as PNA, from wastewater.

The effects of pH on PNA adsorption were investigated (Figure 9b). For the COM–MWCNTs and CO_2_–MWCNTs, the *q_t_* values were almost constant at pH = 2–11, indicating that their adsorption ability was not affected by the pH level. For the KOH–MWCNTs, *q_t_* was lowest at pH = 6. The low adsorption efficiency was because the surface of the KOH–MWCNTs contained OH^−^ ions. The electrostatic attraction between the adsorbent and adsorbate may have been smaller when OH^−^ ions were neutralized with weak H^+^ solution. For the ACID–MWCNT sample, *q_t_* was constant at pH = 2–9 and then decreased as the pH was increased to 11. The surface of the ACID-MWCNTs contained H^+^ ions after acid treatment. When the medium was strongly basic, the nanotubes lost H^+^ ions and did not easily interact with PNA molecules through electrostatic force [49]. The effect of the stirring speed on PNA adsorption was investigated. The samples were stirred at speeds of 50–150 rpm. The adsorption capacity was found to be positively correlated with the stirring speed (Figure 9c). A high stirring speed may promote interaction between PNA and the nanotubes and decrease the thickness of the diffusion layer on the nanotube surface. However, the change in the adsorption capacity was not obvious when the stirring speed was faster than 100 rpm, indicating that mass transfer resistance could be ignored. The effect of the CNT amount on the adsorption capacity was also investigated. A CNT amount of 10–20 mg (Figure 9d) was employed. The adsorption capacity decreased slightly as the CNT amount was increased from 10 to 15 mg. This decrease in the adsorption capacity was due to more adsorption sites being provided when the amount of nanotubes was increased [50]. A further increase in the CNT amount from 15 to 20 mg did not influence the adsorption capacity because the majority of the PNA could already be adsorbed and more PNA was not available for adsorption. Photographs of the residual solutions after degradation of PNA for 150 min are shown in Figure 9e. Visual observation confirmed that the KOH–MWCNTs were a better PNA adsorbent than the CO_2_–MWCNTs or ACID–MWCNTs.

### 3.5. Adsorption Isotherm, Kinetics, and Mechanism

**Figure 10:**
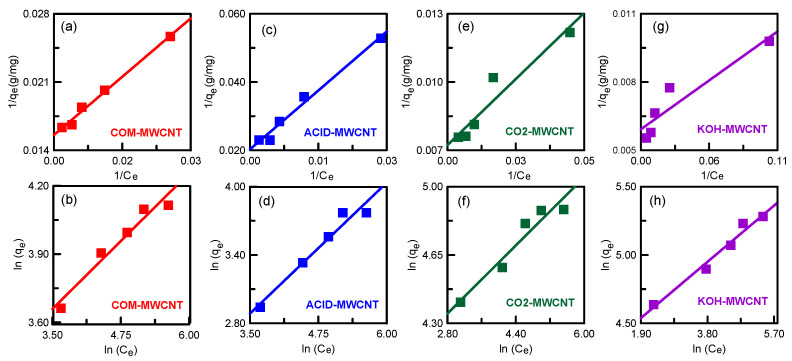
Adsorption isotherms for PNA adsorption onto MWCNTs: (**a**,**c**,**e**,**g**) Langmuir model; (**b**,**d**,**f**,**h**) Freundlich model.

The Langmuir and Freundlich models were employed to acquire information on the PNA distribution between solid and liquid phases at equilibrium. The linear Langmuir and Freundlich equations are represented as follows [51]:

(2) $$\frac{1}{q_{e}}=\frac{1}{q_{L}}+\frac{1}{q_{L}K_{L}C_{e}}$$

(3) $$logq_{e}=logK_{F}+\frac{1}{n}logC_{e}$$

where *n*, *K_L_*, and *K_F_* are the Langmuir and Freundlich constants; *C_e_* (mg/L) is the PNA equilibrium concentration; and *q_e_* and *q_L_* (mg/g) are the equilibrium and maximum adsorption capacity, respectively. The two isotherm models, before and after modification of nanotubes, are plotted in Figure 10. The calculated parameters are listed in Table 3. The determination coefficients R^2^ indicate that the best fit for the COM–MWCNTs and ACID–MWCNTs was the Langmuir model. The model revealed that a single PNA layer uniformly covered the surface of the nanotubes [52]. Conversely, the Freundlich model was the best fit for the CO_2_–MWCNTs and KOH–MWCNTs. The model assumed multilayer adsorption of PNA on the nanotubes with a heterogeneous surface [53]. The separation factors *R*_L_ of the CNTs were in the range 0.1304–0.6329. These values are less than one, suggesting that nanotubes were beneficial to PNA adsorption [54].

**Figure 11:**
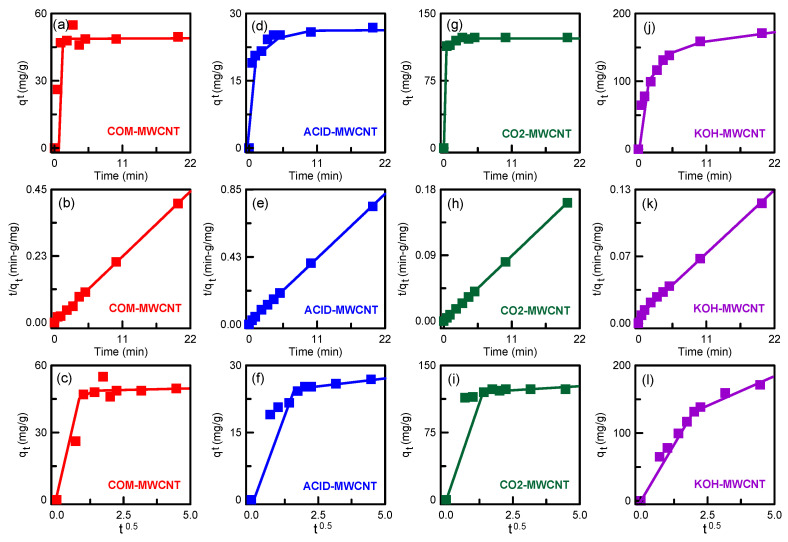
Adsorption kinetics for PNA adsorption onto MWCNTs: (**a**,**d**,**g**,**j**) pseudo-first-order model; (**b**,**e**,**h**,**k**) pseudo-second-order model; (**c**,**f**,**i**,**l**) intraparticle diffusion model.

To understand the PNA adsorption process, pseudo-first-order, pseudo-second-order, and intraparticle diffusion models were used to fit the experimental data. The three models were represented as follows [55]:

(4) $$q_{t}=q_{e}(1-e^{-k_{1}t})$$

(5) $$\frac{t}{q_{t}}=\frac{1}{k_{2}q_{e}^{2}}+\frac{t}{q_{e}}$$

(6) $$q_{t}=k_{i}t^{0.5}+I$$

where *k*_1_, *k*_2_, and *k_i_* are rate constants; *q_t_* and *q_e_* (mg/g) are the adsorption capacity at time *t* and at equilibrium, respectively; and *I* is the boundary thickness. Linear plots of the pseudo-first-order and pseudo-second-order models are shown in Figure 11. For all types of nanotube, the *R*^2^ values clearly indicate that the pseudo-second-order model gives the best fit to the adsorption data (Table 4), indicating chemisorption between PNA and the nanotubes [56]. The experimental *q_e_* values were consistent with the calculated *q_e_* values.

The diffusion mechanism was observed by using an intraparticle diffusion model (Figure 11). Two linearity curves were obtained, indicating that diffusion occurred in two stages. A similar mechanism was discovered by Guo et al. [57] for malachite green adsorption on magnetically activated carbons, which were obtained from peanut shell and FeCl_3_·6H_2_O by CO_2_ activation. In the first stage, PNA molecules diffused into the boundary layer of the nanotubes. In the second stage, the PNA molecules diffused into the pores and then arrived at the surface of the CNTs. The *k_i_* values for the first stage were larger than those for the second stage (Table 4). This was because, in the first stage, the mass transfer resistance was not high enough in the bulk fluid, enhancing PNA diffusion. Over time, the diffusion rate decreased, which may be because diffusion of PNA into the nanotube pores needed a longer duration. The CO_2_–MWCNTs and KOH–MWCNTs had a higher PNA diffusion rate than did the COM–MWCNTs and ACID–MWCNTs (Table 4), indicating less competition for adsorption sites in the activated nanotubes with a high surface area.

The activated nanotubes (CO_2_–MWCNT and KOH–MWCNT) exhibited higher adsorption capabilities than did the pristine and acid-modified nanotubes (COM–MWCNT and ACID–MWCNT, respectively), possibly because the activated nanotubes had a high surface area (Table 2) and more sites available for capturing PNA molecules. Furthermore, PNA and the activated nanotubes had C=C double bonds that contained π electrons [10]. The π electrons favored π–π interactions between the nanotubes and aromatic PNA rings [58]. The activated nanotubes also had a large pore volume, which facilitated electrostatic attraction and van der Waals forces on the surface of the nanotubes and effective interaction between PNA molecules and the nanotubes, thereby increasing the adsorption capacity.

### 3.6. Reutilization Experiments

**Figure 12:**
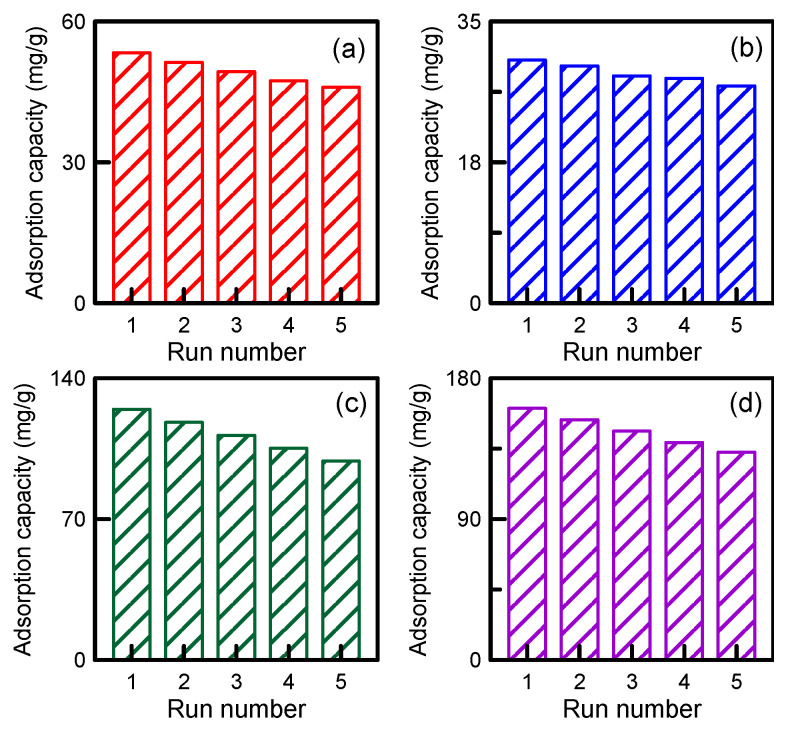
PNA adsorption–desorption experiment for five runs: (**a**) COM-MWCNT, (**b**) ACID-MWCNT, (**c**) CO_2_-MWCNT, and (**d**) KOH-MWCNT.

The cost of nanotube-based wastewater treatment can be reduced by reusing the nanotubes. The adsorption capacities of the COM–MWCNTs, ACID–MWCNTs, CO_2_–MWCNTs, and KOH–MWCNTs after regeneration processes were found to be 86.22%, 89.86%, 79.35%, and 82.58%, respectively (Figure 12). The nanotubes retained very high adsorption capacity after five cycles. The experimental results thus confirmed that the MWCNTs are stable even when reused.

The PNA adsorption performance of the activated nanotubes was compared with that of other materials (Table 5) [1,59,60,61,62,63,64,65,66,67]. Different adsorbents have distinct functional groups. Therefore, a comparison is useful. The activated nanotubes had higher adsorption capacities and shorter equilibrium time in our experiments than did adsorbents reported in other studies. Despite the higher adsorption capacity of the single-walled CNT and activated carbon fiber in the literature, their equilibrium times are longer than that of activated nanotubes in the present study. The mesoporous structure of activated nanotubes can provide relatively large pore size for rapid mass transfer. The combined influence of a high surface area and C–O functional groups on the nanotube surface promoted PNA adsorption.

## 4. Conclusions

This study investigated the use of surface-activated and modified MWCNTs as efficient nanoadsorbents for the removal of PNA from aqueous solutions. The surface area and pore volume were greatest for the KOH–MWCNTs, followed by the CO_2_–MWCNTs, COM–MWCNTs, and ACID–MWCNTs. The appearance of the nanotubes was not obviously changed after acid or alkali modification. The ACID–MWCNTs exhibited the highest thermal stability. The CO_2_–MWCNTs and KOH–MWCNTs exhibited higher PNA adsorption capacities (123.4 and 171.3 mg/g, respectively) than did the pristine and acid-treated nanotubes (49.6 and 26.8 mg/g, respectively). The enhancement of adsorption capacity primarily depends on the increase of pore volume and surface area after surface-activated MWCNTs. The adsorption capacity of the CO_2_–MWCNTs in the initial 2 min was higher than that of the KOH–MWCNTs due to the wide pores of the CO_2_–MWCNTs. The equilibrium data were fitted using the Langmuir model for the COM–MWCNTs and ACID–MWCNTs and the Freundlich model for the CO_2_–MWCNTs and KOH–MWCNTs. For all types of nanotube, the best fit to the kinetic data was achieved using a pseudo-second-order model. CO_2_–MWCNTs and KOH–MWCNTs were found to have several advantages over the other nanoadsorbents, including a high surface area and abundant C–O functional groups. According to our reutilization experiments, the synthesized nanotubes have excellent reusability, with high adsorption capacity (>79%) after five cycles. The activated MWCNTs synthesized in this study have great potential for effectively removing both organic and inorganic pollutants and can be applied on a large scale for wastewater treatment purposes.

## Figures and Tables

**Table 1:** Amounts of metallic impurities in MWCNTs (results in μg/g).

CNTs	Mg	Ca	Fe	Mo	K	Ni	Zn	Al
COM-MWCNT	100	12	137	18	39	21	38	89
ACID-MWCNT	46	ND	77	ND	15	ND	16	32
CO_2_-MWCNT	63	15	125	12	21	15	31	59
KOH-MWCNT	52	10	85	8	215	ND	25	28

ND means not detected.

**Table 2:** Surface area and pore textural parameters of MWCNTs.

Sample	S_BET_ (m^2^/g)	V_t_ (cm^3^/g)	V_mic_ (cm^3^/g)	V_meso_ (cm^3^/g)	V_meso_/V_t_ (%)	d_P_ (nm)
COM-MWCNT	189	1.039	0.011	1.028	98.94	24.06
ACID-MWCNT	80	0.871	0.074	0.797	91.50	36.29
CO_2_-MWCNT	484	1.321	0.009	1.312	99.32	14.87
KOH-MWCNT	487	1.432	0.034	1.398	97.63	10.70

S_BET_ = specific surface area, V_t_ = total pore volume, V_mic_ = micropore volume, V_meso_ = mesopore volume, d_P_ = pore diameter (BJH desorption).

**Table 3:** Parameters of isotherm models for PNA adsorption on MWCNTs.

Sample	Langmuir				Freundlich		
	R_L_	q_L_ (mg/g)	K_L_	R^2^	n	K_F_ (mg/g)	R^2^
COM-MWCNT	0.3455	67.98	0.0344	0.9960	4.1893	16.834	0.9763
ACID-MWCNT	0.6329	57.47	0.0121	0.9921	3.5777	3.6668	0.9780
CO_2_-MWCNT	0.2238	138.31	0.0621	0.9581	4.5777	42.012	0.9645
KOH-MWCNT	0.1304	181.81	0.1298	0.9372	4.6106	61.893	0.9875

**Table 4:** Parameters of kinetic models for PNA adsorption on MWCNTs.

Model	Parameter	Value			
		COM-MWCNT	ACID-MWCNT	CO_2_-MWCNT	KOH-MWCNT
Pseudo-first-order adsorption kinetic	q_e,experiment_ (mg/g)	49.6	26.8	123.4	171.3
	q_e,calculated_ (mg/g)	49.869	24.766	121.70	156.69
	k_1_ (min^−1^)	1.8562	2.4666	5.2611	0.5613
	R^2^	0.9640	0.9612	0.9956	0.9404
Pseudo-second-order adsorption kinetic	q_e,experiment_ (mg/g)	49.6	26.8	123.4	171.3
	q_e,calculated_ (mg/g)	49.850	27.11	123.76	178.89
	k_2_ (min^−1^)	0.1669	0.1236	0.1898	0.0048
	R^2^	0.9994	0.9997	0.9999	0.9968
Intraparticle diffusion kinetic	k_i1_ (mg/g min^1/2^)	24.35	4.74	9.90	50.85
	I_1_	14.50	15.64	106.01	28.13
	k_i2_ (mg/g min^1/2^)	1.08	0.69	0.44	15.96
	I_2_	45.07	23.78	121.74	102.78

**Table 5:** Comparison of adsorption capacity for PNA adsorption onto different nanoadsorbents.

Adsorbent	q_max_ (mg/g)	Equilibrium Time (min)	Adsorbate	Reference
Hyper-crosslinked polymeric resin	110	500	PNA	[1]
γ-Al2O3 nanomaterial	96.85	60	PNA	[59]
Nanographenes	22.8	150	PNA	[60]
Natural coal	41	--	PNA	[61]
Monolithic cryogel disc	160	120	PNA	[62]
Biochar	114	2	PNA	[63]
Bamboo charcoal	170	200	PNA	[64]
Single-walled CNT	204	300	PNA	[65]
Activated carbon	152	250	PNA	[66]
Activated carbon fiber	406	--	PNA	[67]
CO_2_-MWCNT	123.4	5	PNA	Present work
KOH-MWCNT	171.3	20	PNA	Present work

## Data Availability

Data are contained within the article.
